# The Relationship between Resilience and Moral Distress among Iranian Critical Care Nurses: A Cross-sectional Correlational Study

**DOI:** 10.4314/ejhs.v32i2.21

**Published:** 2022-03

**Authors:** Fateme Talebian, Abolfazl Hosseinnataj, Tahereh Yaghoubi

**Affiliations:** 1 Student Research Committee, Mazandaran University of Medical Sciences, Sari, Iran; 2 Faculty of Health, Mazandaran University of Medical Sciences, Sari, Iran; 3 Traditional and Complementary Medicine Research Center, Mazandaran University of Medical Sciences, Sari, Iran

**Keywords:** Resilience, Moral distress, Intensive Care Unit, Nurse, Critical Care Unit

## Abstract

**Background:**

Critical care nurses usually experience high levels of moral distress due to the nature of their work. Resilience in critical care nurses can potentially facilitate effective adaptation to physical and emotional burden of caring for critically ill patients. The present study aimed to evaluate the relationship between resilience and moral distress among Iranian critical care nurses.

**Methods:**

In this cross-sectional correlational study, 144 critical care nurses working in intensive care units (ICUs) of five teaching hospitals affiliated to Mazandaran University of Medical Sciences, were selected randomly, from July to October 2020. Data were collected using a 36-item moral distress questionnaire and a 25-item Connor-Davidson Resilience Scale (CD-RISC) questionnaire. Data were analyzed using independent t-test, ANOVA and Pearson correlation coefficient using SPSS 21 software.

**Results:**

The mean score of moral distress in nurses was 66.93±2.47 and 95.8% of nurses had low-level moral distress. The mean score of resilience in nurses was 90.66±10.92 and 73.6% of nurses had high levels of resilience. There was a statistically significant relationship between the scores of moral distress and resilience. Also, there was a significant positive relationship between work experience and two subscales of resilience as “trust in individual instincts, tolerance of negative emotions” and “impact of spirituality” (p<0.001).

**Conclusion:**

The results of the present study indicated a positive correlation between resilience and moral distress in nurses working in ICUs. It seems that when moral distress increases, critical care nurses increasingly use the mechanism of resilience for better permanence and active presence in the organization.

## Introduction

Ethics, as a main element of nursing profession, includes values and principles that, in certain circumstances, determine the right and wrong of performances (1–3). Nowadays, one of the most common moral issues in the health profession is moral distress. It occurs “when one knows the right thing to do, but institutional constraints make it nearly impossible to pursue the right course of action” (4, 5). Generally, nurses are more likely to develop moral distress than other healthcare professionals such asphysicians, due to the nurse's perceived inability to make decisions and their feeling of being “voiceless” during morally complex conversations (4–6). Moral distress is complex phenomenon. If the experience of moral distress remains unresolved, it can impact the physical, psychological, and emotional wellbeing of nurses (6–7). Moral distress in nurses can actually have negative consequences such as job stress, burnout, reduced job satisfaction, reduced quality of care and ultimately the intention to leave the profession. The highest intention to leave the nursing profession is usually in nurses working in intensive care units (ICUs) and emergency departments. It has been previously indicated that more than 80% of ICU nurses experience moral distress (6). Frequent exposure of critical care nurses to death, involvement in end-of-life discussions, and also stressful working conditions and environment in ICUs are the most important reasons for this intention to leave the profession (6–9). All of these can have detrimental effects on the health care system due to the loss of staff, patient dissatisfaction and increased legal complaints from medical institutions (10, 13).

Resilience is one of the personality traits that can act as a buffer against stressful events and psychological problems caused by work-related stress and help nurses adapt and cope with stress of their work environment (14–16). Resilient nurses would learn to overcome these difficulties and develop better coping mechanisms to address stress through exposure to difficult working situations and environments. Therefore, strengthening resilience skills is one of the potential coping strategies that helps nurses to deal effectively with stressful situations. Strengthening resilience may help reduce moral conflicts (17, 18). Resilience refers to a person's ability to adapt to destructive, stressful, or challenging life events in order to maintain balance against the negative effects of the stress (15,19). Limited studies have evaluated the relationship between resilience and moral distress in nurses. Some studies reported an inverse association between resilience and moral distress in nurses, but this was not confirmed by other studies (20–21).

Considering that moral distress is a major issue in nursing profession and ICU nurses usually experience a great deal of moral distress, as well as paucity of research evidence regarding the potential role of internal characteristics such as resilience to cope with stress and its relationship with moral distress, the aim of this study was to evaluate the relationship between moral distress and resilience in nurses working in ICUs.

## Methods

This descriptive cross-sectional correlational study was conducted on 144 nurses working in the ICUs of five teaching hospitals affiliated to Mazandaran University of Medical Sciences, Sari, Iran, from July to October 2020. Inclusion criteria were having a BSc degree or higher in nursing, work experience in ICU for at least 2 years and willingness to participate in the study. Incomplete questionnaires were excluded and those subjects were not asked on second time to attend the study.

Stratified sampling method was used to select nurses and collect information. For data collection, socio-demographic characteristics questionnaire and moral distress and resilience questionnaires were used. The sociodemographic characteristics of nurses included gender, age, marital status, work experience, work shift, average overtime hours, job position, and employment status. For evaluating nurses' moral distress, the Corley Moral Distress Questionnaire was used. This questionnaire was designed by Corley et al. (22) and Bikmorad et al. confirmed the reliability of this scale among Iranian nurses, with Cronbach's alpha of 0.93 (23). This 36 items questionnaire assesses the severity of moral distress based on nurses' clinical situations using a 6-item Likert scale ranging from *never* (score 1) to *always* (score 6). The total scores of the questionnaire are between 1 and 216, would be categorized as low (1 to 72), moderate (73 to 153) and severe (154 to 216) (22).

The Conor and Davidson Resilience Questionnaire (RISC-CD) was used for evaluating resilience of nurses. This self-reported questionnaire was designed in 2003 (24) and Keyhani et al. assessed it psychometrically in Iran (25). The reliability of the instrument was reported to be 0.93 using Cronbach's alpha. This questionnaire consists of 25 items. It measures 5 components of “perception of individual competence”, “trust in individual instincts”, “endurance of negative emotions”, “positive acceptance of changing safe relationships of control and spiritual effects”. Answers to expressions is based on the five-point Likert scale, in which *completely incorrect* answer gets 1, *rarely correct* 2, *sometimes correct* 3, *often correct* 4, and *always correct* gets 5. In general, the minimum resilience score of the subjects in this scale is 25 and the maximum score is 125. Overall, a score higher than 83 indicates a high degree of resilience (24).

After obtaining permission from the ethical committee of Mazandaran University of Medical Sciences (ethical code IR.MAZUMS.REC.1399.418) and obtaining informed consent from nurses, the questionnaires were randomly distributed in ICUs. Prior to completing the questionnaire, all of the participants who met the inclusion criteria provided informed consent. Questionnaires were anonymous, and no personal identifying data were recorded. Questionnaires were completed and returned by ICU nurses.

**Sample size calculation**: The estimation of sample size was based on a presumed effect size of 0.3, a statistical power of 95%, and a type I error of 5% using G*Power software, version 3.1.3 with the formula for calculation of sample of correlational studies. The overall proper sample size was found to be 144 participants.

**Statistical analysis**: Kolmogorov-Smirnov test was used to investigate the normal distribution of data. As for statistical inference, independent t-test (to compare the mean score of questionnaires in two-level qualitative variables), analysis of variance (ANOVA) [to compare the mean score of questionnaires in slightly more than two-level variables] and Pearson correlation coefficient (correlation between two quantitative variables) were used. Data were analyzed using IBM SPSS 21 software. P-value <0.05 was considered statistically significant.

## Results

In this study, a total of 144 nurses working in ICUs were evaluated. The mean and standard deviation of nurses' age was 32.98 ± 5.63 years. 70.8% of female nurses and 72.9% of all nurses were married. Most nurses had work experience of 10 years or more, or 5 years or less (49.3% and 47.2%, respectively). The mean and standard deviation of the moral distress score in the nurses was 66.93 ± 2.47 (maximum score of 76 and minimum score of 63), which indicates a low level of moral distress in ICU nurses. Of the total participants in the study, 138 nurses (95.8%) had low levels of moral distress and the rest had moderate levels of moral distress. None of the nurses had severe moral distress.

The mean and standard deviation of the resilience scores of the nurses was 90.66±10.92 (maximum score of 99 and minimum score of 65). Approximately 73.6% of nurses had high resilience and the rest of the nurses (26.4%) had moderate resilience. Nurses have the highest mean score (6.81) in the subscale of “*Positive Acceptance of Change and Safe Relationships*” and the lowest mean score (1.75) in the subscale of “*Trust in Individual Instincts and Negative Emotion Endurance*” (Table 1).

**Table 1:** Scores and standard deviation of questionnaires and subscales

Questionnaire	Total score/subscale	Mean	Standard deviation
Moral Distress	Total score	66.93	2.47
Resilience (Dimensions and total score)	Perception of individual competence	13.69	1.22
	Trust in individual instincts, Endurance, negative emotions	8.58	1.37
	Positive acceptance of change and secure relationships	11.81	0.62
	control	4.99	0.57
	spiritual effects	7.21	1.45
	Total score	90.66	10.92

There was a statistically significant relationship between the scores of moral distress and resilience (correlation coefficient: 0.31). As the score of moral distress increases, so does resilience. Table 2 shown the Pearson correlation coefficient between the dimensions of the resilience and moral distress among nurses. According to this table, dimensions of “perception of individual competence”, “trust in individual instincts, endurance, negative emotions”, “positive acceptance of change and secure relationships” and “spiritual effects” in resilience had a significant direct relationship with the moral distress. As the score of each of these dimensions increases, moral distress increases too (Table 2).

**Table 2:** Correlation coefficient of dimensions of resilience questionnaire and moral distress questionnaire

Variable	Perception of individual competence	Trust in individual instincts, Endurance, negative emotions	Positive acceptance of change and secure relationships	Control	Spiritual effects	Moral distress
Perception of individual competence	-	-.02 (.829)	.51 (<.001)	-.07 (.428)	.78 (<.001)	.73 (<.001)
Trust in individual instincts, Endurance, negative emotions		-	-.21 (.010)	-.39 (<.001)	-.48 (<0.001)	.45 (<.001)
Positive acceptance of change and secure relationships			-	.27** (.001)	.56 (<.001)	.59 (<.001)
Control				-	-.03 (.716)	.06 (.480)
Spiritual effects					-	.38 (<.001)

Table 3 revealed the effects of variables on each of the scores of moral distress and resilience by linear regression. According to the results, only work experience had a significant relationship with resilience, so that nurses with 6 to 9 years of experience had a lower resilience score (19.34 points) than nurses with less than 6 years of experience.

**Table 3:** the effect of demographic variables on nurses 'moral distress and resilience score based on linear regression

Variable		Resilience score			Moral distress score		
		
		B	SE	p-value	B	SE	p-value
Gender	Male	-	-	.401	-	-	**.208**
	Female	.39	.46		2.44	1.93	
Marital status	Single	-	-	.255	-	-	**.136**
	Married	.55	.48		3.04	2.02	
Work experience	<6	-	-	-	-	-	-
(years)	6–9	.43	1.28	.736	-19.34	5.39	**<.001**
	>9	-.31	.56	.576	-1.48	2.36	**.531**
Age (years)	<31	-	-	-	-	-	-
	31–40	-.56	.54	.307	-1.52	2.28	**.507**
	>40	-.58	.80	.471	4.02	3.36	**.234**

## Discussion

This study investigated the relationship between resilience and moral distress among Iranian ICU nurses. The present study showed that most nurses working in ICU experienced low levels of moral distress. Shafipour et al. conducted a study on the severity of moral distress and its related factors among nurses working in the burn center. Their findings showed that the majority of nurses experienced moderate levels of moral distress (26) that is not consistent with the findings of present study. Although the type of questionnaire used to assess moral distress is the same in these two studies, working in the burn units, due to the special conditions of patients, exposes nurses to high psychological stress (27) which potentially can lead to higher levels of moral distress in nurses working in these units. In another study conducted by Behbodi et al., the findings showed that 58.8% of nurses working in pediatric ICUs had low levels of moral distress and 41.2% had moderate levels of moral distress; this is somewhat similar to the findings of the present study (28). Also, the results of a study by Vaziri et al. revealed a moderate to high levels of moral distress among Iranian nurses (29).

The present study showed that the majority of nurses working in the ICU had high resilience (73.6%) and the rest of the nurses had moderate resilience. In a study by Mahdieh et al. it has been shown that the average resilience of hospital nurses was moderate (30), while in the present study the average resilience was higher. Consequently, despite the similarity of the questionnaire used to assess the resilience of nurses, the findings are not consistent with our study, which may be due to the specific working conditions of ICUs in the present study.

Moreover, the psychological and behavioral characteristics of nurses and also their knowledge and culture can strongly affect their resilience levels (15). The results of a study by Gerami Nejad et al. also showed that the resilience scores of nurses in ICUs are almost similar to the present study (31). The results of a study that investigated the relationship between demographic characteristics and resilience of nurses in Singapore showed that higher resilience correlated with marital status, age, higher education and job position (32). In the present study, only work experience has a significant relationship with resilience. This discrepancy can be due to different working and cultural conditions in the two populations.

A study on the United States' emergency nurses revealed that nurses' resilience increases with the increased age and their job satisfaction. In addition, they showed that there is no relationship between moral distress and resilience (33). The results of a study indicate that ICU nurses usually use more resilience in the moral distress to be able to fulfill their obligations in the workplace (34). The results of another study showed that higher moral resilience in healthcare professionals is associated with a lower burnout and turnover intentions (35).

In a study on the female nurses in China, Zou et al. found that 85.5% of them experienced psychological distress and there was a negative relationship between psychological distress and resilience. So that with increasing moral resilience, the levels of psychological distress decreased. The mediating role of resilience between psychological distress and burnout was also confirmed (36). The results of a study by Abdollahi et al. indicate a positive and significant relationship between resilience and professional moral courage in nurses (37). Considering that the competing priorities and challenges with which nurses are confronted may make it difficult to develop resilience characteristics, it's important for nurse leaders to educate nurses about techniques to build resilience. This study have some limitation. Considering that the statistical population of this study was mostly female critical care nurses who worked in teaching hospitals, therefore it may hamper generalizability of the results. Also, considering the Covid-19 pandemic, psychological conditions of nurses at the time of completing the questionnaires could affect the results of the study, which was beyond the control of the researchers. In addition, the nature of the cross-sectional design of the current study limits our ability to form firm conclusions regarding causality.

In conclusion, the results of this study revealed a significant relationship between resilience and moral distress among ICU nurses. Therefore, it seems that when moral distress increases, critical care nurses increasingly use the mechanism of resilience for permanence and active presence in the organization. By using formal education and social support nurse leaders can contribute to building nurse resilience to establish a healthy work environment and maintaining a stable nurse workforce.
